# Next-Generation Sequencing in Differentiated Thyroid Cancer Patients Treated with Lenvatinib: Results and Challenges in Real-Life Practice

**DOI:** 10.3390/curroncol33060372

**Published:** 2026-06-21

**Authors:** Matteo Ferrari, Alice Nervo, Francesca Maletta, Sara Mariani, Elisa Vaccaro, Alessandro Piovesan, Emanuela Arvat

**Affiliations:** 1 Oncological Endocrinology Unit, Città Della Salute E Della Scienza Hospital, Department of Medical Sciences, University of Turin, Via Genova 3, 10126 Turin, Italy; matteo.ferrari@aslcn1.it (M.F.); anervo@cittadellasalute.to.it (A.N.);; 2 Division of Diabetology and Metabolic Disease, Department of Medicine, Azienda Sanitaria Locale Cuneo 1, 12100 Cuneo, Italy; 3 Pathology Unit, Città Della Salute E Della Scienza Hospital, 10126 Turin, Italy; 4 Endocrine and Metabolic Diseases Unit, Cardinal Massaia Hospital, 14100 Asti, Italy

**Keywords:** thyroid carcinoma, targeted therapy, molecular profiling, next-generation sequencing

## Abstract

Objective: Our objectives were to describe molecular profiling in a real-life cohort of patients with radioiodine-resistant (RAI-R) differentiated or poorly differentiated thyroid cancer (DTC or PDTC) treated with lenvatinib and to focus on factors potentially influencing the quality of tissue samples for molecular analysis, including the impact of storage time, defined as the interval between tissue collection and molecular testing. Design: We retrospectively included all lenvatinib-treated RAI-R DTC or PDTC patients tested with DNA- and/or RNA-based next-generation sequencing (NGS) in our center, also analyzing the results of fluorescence in situ hybridization (FISH) for RET fusions if the sample did not satisfy quality criteria for RNA-based NGS analysis. We investigated differences in terms of histotype, biopsy site, or storage time between adequate and inadequate samples for RNA-based NGS. Results: At least one gene alteration was detected in 50% of the cohort (18 out of 36 patients); RAS and BRAF were the most frequent mutations, while gene fusions accounted for 5.6% of cases. Tissue samples were more frequently adequate for DNA-based NGS compared to RNA-NGS analysis (93.9% vs. 58.3%, *p* < 0.001). The median storage time was significantly longer in the case of inadequate samples for RNA-based NGS compared with adequate specimens (41.5 vs. 9.5 months, *p* = 0.016); samples archived for ≥3 years led more frequently to an inadequate result. Conclusions: Advanced RAI-R TC candidates for systemic therapy often harbor gene alterations. An adequate result was less frequently achieved in cases of RNA-based NGS than in DNA-based NGS, especially if the interval between tissue collection and molecular analysis was longer; nevertheless, the limited cohort size precludes definitive conclusions.

## Simple Summary

This study describes the molecular profiling of a real-life cohort of patients with radioiodine-resistant differentiated or poorly differentiated thyroid cancer treated with lenvatinib. The research found that 50% of patients harbored at least one gene alteration, with RAS and BRAF mutations being the most frequent, followed by gene fusions. A key focus of the study was the quality of tissue samples used for Next-Generation Sequencing (NGS). Results showed that DNA-based NGS was significantly more successful than RNA-based analysis (93.9% vs. 58.3%). Notably, the storage time of tissue samples played a critical role; samples archived for three years or longer frequently led to an inadequate RNA results. These findings emphasize that the timing of tissue collection is vital for successful molecular characterization, particularly for identifying gene fusions.

## 1. Introduction

Approximately 10% of patients with differentiated thyroid cancer (DTC) develop distant metastases, and up to one-third of these cases become radioiodine-resistant (RAI-R), frequently requiring systemic treatment with multikinase inhibitors (MKIs) [1].

Lenvatinib, a MKI targeting vascular endothelial growth factor (VEGFR) 1–3, fibroblast growth factor receptor (FGFR) 1–4, platelet-derived growth factor (PDGFR) α, rearranged during transfection (RET) and KIT is approved by the Food and Drug Administration (FDA) and the European Medicines Agency (EMA) as a first-line therapy for patients with progressive, locally advanced and/or metastatic RAI-R DTC [2].

Both in the registration trials and in the subsequent real-life studies, the high incidence of adverse events (AEs) and the onset of acquired resistance have emerged as major lenvatinib-associated issues, frequently limiting the continuation of treatment [3,4,5,6].

Gene alterations leading to activation of the mitogen-activated protein kinase (MAPK) pathway are crucial for tumorigenesis and progression in DTC [7]. In recent years, molecular profiling has gained increasing clinical relevance in advanced DTC, enabling the identification of patients eligible for gene-specific targeted therapies, especially those directed against RET, neurotrophic tyrosine receptor kinase (NTRK), anaplastic lymphoma kinase (ALK) or ^V600E^BRAF alterations [8]. These emerging therapeutic approaches have demonstrated high efficacy and low toxicity in this setting [9,10,11]. For RET, NTRK or ALK-altered RAI-R DTC tumours, the use of selective inhibitors is recommended even in first-line setting according to the recent ATA guidelines [12]. When feasible, they all represent a valuable strategy for poor candidates for MKI as well as for patients who are intolerant to or have progressed on one or more prior MKI therapy [12]. 

Other gene alterations, such as the mutation of TP53 or telomerase reverse transcriptase (TERT) promoter, are recognized as negative prognostic factors, since they are associated with increased tumor aggressiveness and diminished responsiveness to lenvatinib when associated with a driver mutation, albeit with some conflicting findings in the literature [13,14,15]. Therefore, a comprehensive molecular analysis may provide valuable information for more accurate disease characterization [16,17].

According to the recent ATA guidelines [12], the latest European Society for Medical Oncology (ESMO) recommendations [8], and the American consensus endorsed by the Endocrine Surgery Section of the American Head and Neck Society (AHNSESS) and the International Thyroid Oncology Group (ITOG) [18], molecular testing should be offered to patients with progressive disease prior to initiating systemic therapy. Next-generation sequencing (NGS), preferably DNA- and RNA-based, should be used to detect point mutations and gene rearrangements.

Although molecular profiling has gained clinical utility, offering patients the possibility to be treated with selective inhibitors, the timing and modalities of molecular testing remain unstandardized in clinical practice, since they are influenced by the expertise of the local laboratory and their financial resources [18]. Therefore, access to targeted therapies is currently heterogeneous across institutions and countries [19].

NGS allows rapid sequencing of large genomic regions—or even the entire genome—by simultaneously reading millions of DNA fragments. It can detect a wide range of gene alterations, including base substitutions, insertions, deletions, copy number variations, and gene rearrangements. Additionally, it can be used to assess gene expression changes and epigenetic modifications [20]. However, both DNA- and RNA-based NGS platforms have some limitations. DNA sequencing is limited by the presence of non-coding mutations, epigenetic effects, and challenges in detecting gene fusions due to long intronic regions that are often difficult to amplify. Conversely, RNA sequencing is generally considered the gold standard for detecting gene fusions, but it is more vulnerable to pre-analytical factors, which frequently compromise sample adequacy [21,22].

Among pre-analytical variables, storage time—the interval between tissue collection and molecular analysis—is one the most critical, as DNA and RNA yield can decrease over time. Studies have shown that DNA quantity can decline by 47% after six years of storage, with only 11% of the material remaining amplifiable [23]. The effect of storage time appears even more pronounced for RNA [22]. For all these reasons, the application of validated criteria for the evaluation of quality parameters and selecting the right specimen is essential in molecular diagnostics.

If an archival specimen is inadequate for NGS testing, a surgical or core needle biopsy (CNB) is recommended instead of the measurement of circulating tumor DNA (ctDNA), since the role of the liquid biopsy has still to be validated in the setting of RAI-R DTC [12]. However, the clinical feasibility of a new biopsy could be a matter of debate in some cases.

The aim of this study was to describe the results of molecular profiling in a real-life cohort of DTC patients treated with lenvatinib at a single institute, focusing on potential factors influencing the quality of tissue samples for molecular analysis, including the effect of storage time.

## 2. Materials and Methods

We retrospectively analyzed all patients who started treatment with lenvatinib for advanced RAI-R DTC or poorly differentiated thyroid carcinoma (PDTC) at the Oncological Endocrinology Unit of Città Della Salute e Della Scienza Hospital, between 1 January 2015 and 1 November 2025. The analysis included only subjects tested with DNA and/or RNA-based NGS for clinical purposes at our center, either before or during systemic therapy, excluding those tested in external laboratories, to ensure a homogeneous cohort.

Clinical data were retrieved from electronic medical records and entered into a dedicated database. Collected variables included socio-demographic, disease-, and treatment-related data. Radiological response to lenvatinib was assessed according to Response Evaluation Criteria in Solid Tumors (RECIST) version 1.1.

Data regarding both the molecular analysis and the corresponding biological specimens were recorded. Collected variables included the anatomical site of tissue sample and the storage time, defined as the interval between tissue collection and molecular testing.

Mutations and gene fusions were investigated using formalin-fixed paraffin-embedded (FFPE) tissues obtained either from surgical specimens or biopsy samples.

Collected samples were required to meet the morphological eligibility criteria adopted by the Pathology Department of our hospital (>100 viable cells, >50% of neoplastic cells in tissue section). Molecular analysis was performed after semi-automated extraction of genomic DNA and/or RNA from FFPE samples (Maxwell RSC DNA and/or RNA FFPE, Promega, Milano, Italy), followed by real-time PCR analysis of sample concentration and fragmentation. NGS was performed on DNA using the Myriapod NGS Cancer Panel DNA, Diatech Pharmacogenetics, Jesi, Italy, with sequencing conducted on iSeq 100 or MiSeq platforms (Illumina Inc., San Diego, CA, USA or Myriapod NGS Cancer Probe Plus, Diatech Pharmacogenetics, Jesi, Italy). For RNA-based NGS, the Myriapod NGS Cancer Panel RNA kit, Diatech Pharmacogenetics, Jesi, Italy was used and sequencing permormed on the iSeq 100 or MiSeq platforms. Data were analyzed using the Easy PGX Data Analysis Software RT800-5W v. 4.0.0, Diatech Pharmacogenetics, Jesi, Italy.

DNA adequacy assessment was based on mean cluster depth, percentage of uniformity, and reads on target (and percentage of low-coverage exonic bases only in Myriapod NGS Cancer Probe Plus). RNA adequacy assessment was based on cluster mean depth, reads on target (number and percentage), and on the absolute number of reads in three house-keeping control genes. For both DNA and RNA, adequacy was determined by automated analysis. A sample was considered inadequate if it failed to meet even a single criterion. For both DNA and RNA NGS analysis, adequacy was conducted by Myriapod NGS Plus Data Analysis Software cod. NG900-SW v.5.0.9, Diatech Pharmacogenetics, Jesi, Italy.

If the sample did not satisfy quality criteria for RNA-based NGS analysis, we analyzed the results of fluorescence in situ hybridization (FISH) performed using probes for RET (ZytoLight Dual Color Break Apart Probe–ZytoVision GmbH, Bremerhaven, Germany) and ALK fusions (Vysis Break Apart FISH Probe Kit–Abbott, Abbott Park, IL, USA).

Moreover, we investigated potential factors that might have influenced the quality of samples for molecular analysis, including storage time, histotype, and sample site.

The baseline characteristics of the patients included in the analysis are summarized descriptively using the median and interquartile range (IQR) or number and percentages. Categorical variables were compared between groups using Fisher’s exact test. Continuous variables were compared between two groups using the Mann–Whitney U test and among multiple groups using the Kruskal–Wallis test. A *p*-value < 0.05 was considered statistically significant. Statistical analyses were performed using GraphPad Prism 10 (GraphPad Software, LLC, Boston, MA, USA).

All procedures performed were in accordance with the ethical standards of the institutional research committee and with the 1964 Helsinki Declaration and its later amendments or comparable ethical standards. The protocol was approved by the Institutional Ethics Committee.

## 3. Results

**Figure 1:**
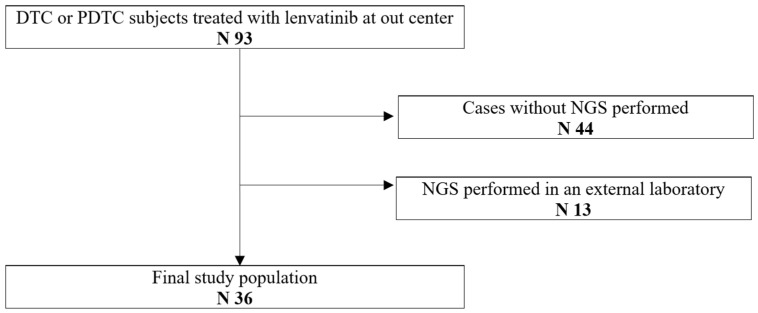
Inclusion and exclusion criteria of the study population; Abbreviations: DTC: differentiated thyroid cancer; NGS: next-generation sequencing; PDTC: poorly differentiated thyroid cancer.

A total of 36 patients were included in the analysis (Figure 1). The main clinical-pathological data of the cohort are displayed in Table 1. Most patients were female (55.6%), and the most represented histotype was follicular thyroid carcinoma (FTC, 38.9%); the disease was metastatic at diagnosis in 38.9% of cases. In the subgroup of patients treated with RAI (72.2%), the median administered activity of ^131^I was 310 mCi (IQR 200–638 mCi). Some 8.3% of the subjects had been pre-treated with another MKI, sorafenib in all cases.

Lenvatinib was started after a median time of 41.9 months (IQR 7.7–69.8) from the initial diagnosis; only 30.6% of the population was started on the recommended dose of 24 mg/day. Median age at lenvatinib initiation was 67 years (IQR 58–74.3), and the most frequent sites of distant metastasis were lung (72.2%) and bone (55.6%).

Radiological assessment during lenvatinib therapy was unavailable in 8.3% of cases. The best response within the first 6 months of treatment was partial response (PR) in 47.2% of patients and stable disease (SD) in 27.8% of subjects; progressive disease (PD) was recorded in the remaining cases. The median follow-up duration from the start of lenvatinib was 33 months (IQR 11.5–65.3). During TKI treatment, radiological PD according to RECIST criteria was observed in 55.6% of cases.

In patients whose NGS was carried out within 2021 (representing 55.6% of the cohort), the analysis was performed after the start of lenvatinib in 88.9% of cases; conversely, the molecular profiling was requested before the initiation of lenvatinib in 60% of subjects whose analysis was performed thereafter.

**Figure 2:**
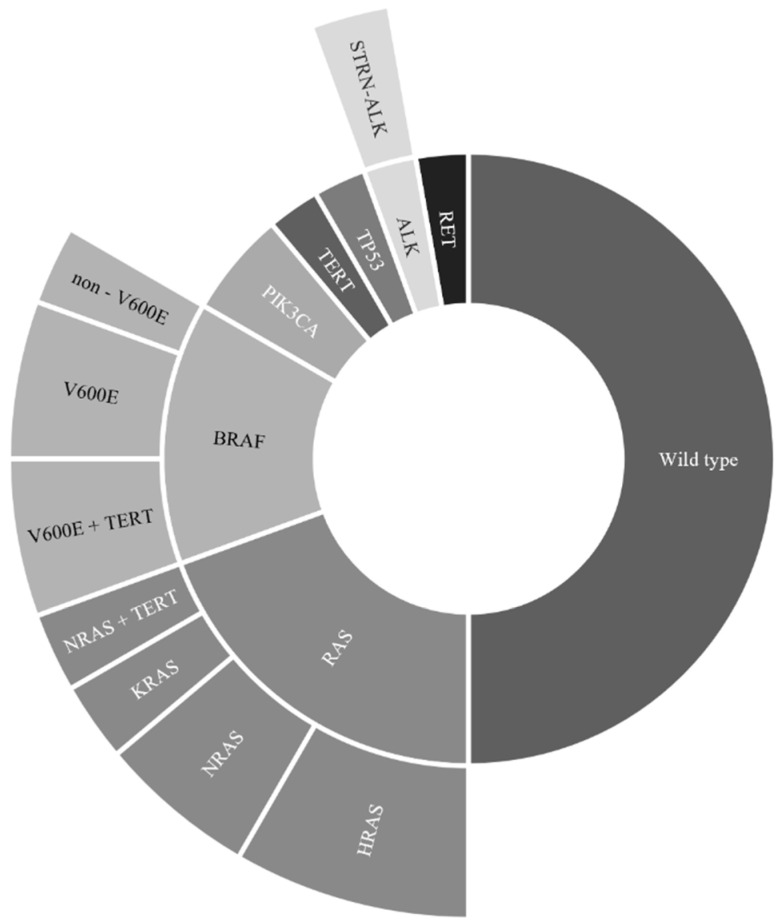
Distribution of gene alterations in our cohort. Abbreviations: ALK: anaplastic lymphoma kinase; PIK3CA: phosphatidylinositol-4,5-bisphosphate 3-kinase catalytic subunit-α; RET: rearranged during transfection; STRN: striatin; TERT: telomerase reverse transcriptase.

Overall, 50% of the study population harbored at least one gene alteration (Figure 2). As displayed in Table 2, the most frequently observed mutations involved RAS (19.4% of the cohort) and BRAF (13.9%). TERT promoter mutation was found in 11.1% of the entire cohort; however, the NGS panel that included also the detection of this specific alteration was employed only in a minority of subjects (6 out of 36 cases). Gene fusions were detected only in two cases (5.6%): one ALK rearrangement revealed by NGS-based RNA, and one RET fusion identified by FISH test in a subject whose NGS-based analysis was inadequate. In the latter patient, this critical finding enabled a therapeutic switch from lenvatinib to the selective RET inhibitor selpercatinib.

DNA-based NGS was performed in 33 out of the 36 patients, with all but 2 samples adequate for the analysis (93.9%). RNA-based NGS was carried out in all subjects; however, the specimen quality was considered insufficient to perform such analysis in more than half of cases (58.3%). RNA-based NGS had a significantly lower rate of adequate results than DNA-based NGS (*p* < 0.001). All patients with failed RNA-NGS were tested for RET fusions using FISH; among these cases (*n* = 21), as reported above, an RET alteration was detected only in one subject (4.8%).

As shown in Table 3, no significant differences were found in terms of histotype or biopsy site between adequate and inadequate samples for RNA-based NGS. In contrast, the median storage time was significantly longer in inadequate samples compared with adequate specimens (41.5 vs. 9.5 months, *p* = 0.016). In particular, the FFPE tissue was archived for ≥3 years in 57.1% of patients whose RNA-based NGS led to an inadequate result, in comparison to 20% of subjects whose analysis was successful (*p* 0.04).

## 4. Discussion

In this real-life study, we analyzed the results of molecular profiling performed for clinical purposes in a cohort of DTC and PDTC patients treated with lenvatinib and followed up at our center. We focused on the practical challenges encountered in routine clinical practice, particularly the impact of sample storage time. This aspect represents a critical issue in slowly progressive malignancies like DTC, where tissue samples are frequently collected months or even years prior to molecular analysis.

At least one gene alteration was found in half of the cohort, with RAS and BRAF being the most frequently observed mutations. Overall, the driver mutation rate in our cohort was lower than previously reported [19,24,25]. The low prevalence of BRAF mutation might be partially explained by the limited number of papillary thyroid carcinoma (PTC) included in our cohort, along with histopathological predominance of follicular thyroid carcinoma (FTC), in which the RAS mutation rate within is consistent with previous series [25,26]. Regarding this aspect, the geographical and nutritional background of our population—characterized by past chronic iodine deficiency—might account for the lower-than-expected rate of classic MAPK pathway drivers, which are reported to be more frequent in iodine-sufficient areas [27].

According to the recent ATA guidelines [12], in RAI-R DTC patients, ^V600E^BRAF-directed therapy may be considered even as a first-line therapy in poor candidates for lenvatinib harboring the ^V600E^BRAF mutation, as well as upon progression and/or intolerance to prior MKI therapy. Similarly to other countries, such targeted treatment is not currently reimbursed for this indication in Italy; however, it must be considered that eligible patients might potentially access novel therapies through basket clinical trials, managed access programs, or off-label use [19].

Targetable gene fusions were detected in 5.6% of the study population, in line with data reported in two European retrospective studies [7,19] and lower than those reported in an Asian multicenter study [26] including RAI-R TC. Although rare, these molecular alterations provide the rationale for employing gene-specific targeted therapy, as recently suggested even in the first-line setting [12]. In our population, the identification of an RET fusion enabled a therapeutic switch from lenvatinib to the selective RET inhibitor selpercatinib.

The preferred analytical method for molecular analysis in DTC is still a matter of debate. According to recent recommendations, testing should be offered in advanced DTC, preferably using NGS techniques on DNA and/or RNA to detect point mutations and gene rearrangements [12,28].

Oncogenic drivers such as RET or NTRK fusions and BRAF mutation are mutually exclusive, and they occur at varying frequencies across TC histotypes. Therefore, some authors suggest a stepwise histology-driven molecular testing strategy to maximize the likelihood of identifying actionable targets and to facilitate access to selective treatments [7,19]. It might be appropriate to initially perform a BRAF single-gene analysis, followed by NGS in BRAF wild-type patients, for assessing targetable gene fusions and identifying candidates for precision therapy [19]. In our center, immunohistochemistry (IHC) for the ^V600E^BRAF mutation is routinely performed prior to NGS only in anaplastic thyroid carcinoma to ensure a faster turnaround time.

In clinical practice, specimens must be adequate to ensure the reliability of the molecular profiling [21]. However, the detection of gene fusions in RNA isolated from FFPE tumor tissues is challenging for technical reasons. The pre-analytical phase is critical for ensuring material suitability for subsequent molecular analysis. Key factors include ischemia during surgery, the post-excision period until formalin fixation, and paraffin embedding. Even brief ischemia can reduce transcription and alter RNA levels; furthermore, RNA is highly susceptible to endogenous enzymatic degradation until formalin fixation is complete [29,30]. Decalcification procedures in bone metastasis specimens may also detrimentally influence RNA quality [31].

Accordingly, in our cohort, DNA-based NGS analyses were more frequently successful than RNA-based NGS tests. A longer sample storage time appeared to significantly reduce the quality of specimen for RNA-based NGS: the FFPE tissue archived for ≥3 years yielded a high rate of inadequate results (57.1% versus 20%). Similar results have been described in other settings. A recent multi-institutional Italian study tested the quality status of stored FFPE blocks and evaluated the proper preservation of archived samples [32]. It was reported that an appropriate DNA quantity was found in 94.4% of the sample, whereas appropriate DNA quality was found in only 62.3% of the cases due to significantly increased fragmentation after 6–8 years of storage. Interestingly, while RNA quantity was optimal across the entire set of cases, RNA quality was adequate only in 22.3% of specimens, with particularly poor data observed in the older samples.

In non-small cell lung cancer (NSCLC), it was reported that a significantly higher proportion of RNA-based NGS analyses failed when performed more than 30 days after tissue sampling [22]. In RAI-R TC and ATC patients, it was recently shown that a longer interval from FFPE tissue inclusion to NGS testing significantly increased the rate of non-evaluable tests, which was higher in specimens stored for more than 5 years [19].

In cases of non-evaluable NGS results, a surgical or core biopsy is recommended, since liquid biopsy remains to be validated in the setting of RAI-R DTC [12]. However, a new biopsy to detect actionable alterations might not be feasible and, in those cases, an alternative single-gene testing approach may be considered [19]. In our cohort, FISH for RET fusions was able to detect a rearrangement in one patient, whose RNA-based NGS had failed. This observation is consistent with a previous study conducted on NSCLC samples, where FISH was effective in detecting gene fusions even in cases of poor quality of specimens for RNA-based NGS analysis [33]. However, the limitations of FISH included the ability to detect only one fusion at a time as well as the risk of false negative results [34].

We did not find any NTRK rearrangements, whose prevalence is low in large-scale studies [35,36,37] and more common in subjects exposed to radiation exposure or in pediatric PTC [38]. However, if the specimen was not adequate for RNA-based NGS, we did not routinely employ alternative approaches for the detection of NTRK fusions; this might have reduced the chance to detect such alterations in our cohort. We found TERT promoter mutations only in 11.1% of the entire population, which is apparently lower than expected in patients with advanced DTC or PDTC [14]. However, it is critical to note that the DNA-based NGS panel including TERT promoter analysis was utilized only in a minority of subjects (6 out of 36 cases). Notably, within this specific subgroup, all but two patients harbored this genetic alteration. According to recent guidelines, molecular testing should be offered in advanced DTC before starting systemic therapy [8,12]. In line with this indication, the rate of molecular analyses requested before the start of systemic therapy increased over time in our center, reducing at least in part the interval from FFPE tissue inclusion to NGS testing and the potential risk of obtaining an inadequate result. However, in our population, lenvatinib was started after a median time of 41.9 months from initial diagnosis, and storage time was ≥3 years in 41.7% of cases.

Our study has several limitations. Firstly, its retrospective design and limited number of patients included in the analysis reduced statistical power, particularly in estimating the true prevalence of gene alterations in the RAI-R DTC setting or in exploring the relationship between mutational status and radiological response to lenvatinib.

Due to the small sample size, we cannot perform a multivariate analysis to better determine the influence of specimen storage time on tissue quality for RNA-based NGS. Moreover, we chose to exclusively report the best objective response during lenvatinib—which was consistent with the literature [2]—rather than deeply exploring survival metrics, as statistical power was insufficient to yield meaningful additional insights on this topic.

However, it must be underlined that the prevalence of advanced DTC requiring systemic therapy is lower than that of other malignancies, in which molecular profiling is routinely employed to guide the selection of targeted therapies. Unlike other studies regarding RAI-R TC submitted to NGS analysis, we included only patients treated with lenvatinib before or after the molecular assessment; therefore, all cases had advanced disease that potentially could gain meaningful clinical benefit from therapy with a selective agent.

## 5. Conclusions

In conclusion, our analysis offers a real-world overview of molecular results from a single referral center, encompassing the current challenges encountered by clinicians in this setting. Advanced RAI-R TC candidates for systemic therapy harbor a notable prevalence of gene alterations, and molecular profiling could provide valuable information in guiding personalized treatment decisions in several cases. Therefore, close collaboration between the clinician, the pathologist, and the molecular biologist is essential to integrate this analysis into routine clinical practice. An adequate result is less frequently achieved in RNA-based NGS than in DNA-based NGS, especially if the interval between tissue collection and molecular analysis is longer. In patients with persistent or recurrent metastatic disease, our results support the opportunity to perform the NGS analysis earlier, prior to the need for systemic therapy. Sample storage time should be considered in selecting the most suitable method for molecular profiling and in deciding whether to attempt a new biopsy. Nevertheless, the limited cohort size precludes definitive conclusions, and our findings require validation in larger prospective studies.

## Figures and Tables

**Table 1:** Main demographic and clinical data of the population.

N (%)	
	**Sex**
20 (55.6%)	Female
16 (44.4%)	Male
	**Histotype**
14 (38.9%)	Follicular
8 (22.2%)	Oncocytic
3 (8.3%)	Poorly differentiated
11 (30.6%)	Papillary
	**Distant metastasis at diagnosis**
22 (61.1%)	No
14 (38.9%)	Yes
	**Distant metastasis before the start of lenvatinib**
20 (55.6%)	Bone
7 (19.4%)	Brain
3 (8.3%)	Liver
26 (72.2%)	Lung
16 (44.4%)	Mediastinum
	**Radioactive-iodine before lenvatinib**
10 (27.8%)	No
26 (72.2%)	Yes
	**Tyrosine kinase inhibitor before lenvatinib**
33 (91.7%)	No
3 (8.3%)	Yes (sorafenib)
	**Lenvatinib starting dose (mg/day)**
11 (30.6%)	24
14 (38.9%)	20
6 (16.7%)	14
5 (13.9%)	10
	**Best response during lenvatinib**
6 (16.7%)	Progressive disease
17 (47.2%)	Partial response
10 (27.8%)	Stable disease
3 (8.3%)	Not available

**Table 2:** Clinico-pathological and molecular data of patients with gene alterations.

Gene Alteration/s	Timing of Molecular Profiling	Storage Time (Months)	Sample Site	Histology	Age * (Years)	Sex	ID
V600E BRAF	After LEN start	8.1	Brain	PTC	73	F	# 1
V600E BRAF	After LEN start	32.6	Subcutaneous	PTC	85	F	# 2
V600E BRAF TERT promoter	Before LEN start	47.5	Primary site	PTC	73	F	# 3
V600E BRAF TERT promoter	Before LEN start	2.5	Primary site	PTC	77	F	# 4
Non-V600E BRAF	After LEN start	112.6	Bone	FTC	57	M	# 5
HRAS	Before LEN start	10	Bone	FTC	59	F	# 6
HRAS	After LEN start	8.5	Bone	PTC	66	M	# 7
HRAS	After LEN start	18.7	Primary site	FTC	70	F	# 8
KRAS	After LEN start	91.5	Lung	PTC	77	M	# 9
NRAS	After LEN start	194.5	Primary site	PDTC	74	F	# 10
NRAS	After LEN start	24	Primary site	PDTC	60	F	# 11
NRAS TERT promoter	Before LEN start	32.9	Lymph node	FTC	62	M	# 12
RET fusion	After LEN start	40.9	Subcutaneous	PDTC	72	M	# 13
STRN-ALK fusion	After LEN start	17	Subcutaneous	PTC	54	F	# 14
PIK3CA	After LEN start	44.1	Primary site	PTC	55	F	# 15
PIK3CA	Before LEN start	9.5	Primary site	PDTC	75	M	# 16
TP53	Before LEN start	8.2	Primary site	OTC	40	F	# 17
TERT promoter	After LEN start	16.1	Lymph node	OTC	79	M	# 18

* At the start of lenvatinib. Abbreviations: ALK: anaplastic lymphoma kinase; F: female; FTC: follicular thyroid cancer; LEN: lenvatinib; M: male; OTC: oncocytic thyroid cancer; PDTC: poorly differentiated thyroid cancer; PIK3CA: phosphatidylinositol-4,5-bisphosphate 3-kinase catalytic subunit α; RET: rearranged during transfection; STRN: striatin; PTC: papillary thyroid cancer; TERT: telomerase reverse transcriptase.

**Table 3:** Differences in histotype, biopsy site, and storage time of samples employed for RNA-based NGS according to the result of the molecular analysis (adequate versus inadequate results). Abbreviations: FTC: follicular thyroid cancer; OTC: oncocytic thyroid cancer; PDTC: poorly differentiated thyroid cancer; PTC: papillary thyroid cancer.

		Adequate Samples N 15	Inadequate Samples N 21	*p*
**Histotype–n (%)**				0.39
	PTC	7 (46.7%)	4 (19.1%)	
	FTC	4 (26.7%)	10 (47.6%)	
	OTC	1 (6.6%)	2 (9.5%)	
	PDTC	3 (20%)	5 (23.8%)	
**Sample site–n (%)**				0.33
	Bone metastasis	1 (6.6%)	5 (23.8%)	
	Other metastasis	7 (46.7%)	6 (28.6%)	
	Primary site	7 (46.7%)	10 (47.6%)	
**Storage time (months)–median (IQR)**		9.5 (2.6–32.8)	41.5 (18.7–72.9)	0.016
**Storage time (months)**				0.04
	<3 years	12 (80%)	9 (42.9%)	
	≥3 years	3 (20%)	12 (57.1%)	

## Data Availability

The data presented in this study are available on request from the corresponding author.
